# Species composition of blow flies (Diptera: Calliphoridae) colonizing pig carcasses exposed to bifenthrin‐ and clothianidin‐containing products

**DOI:** 10.1111/mve.70063

**Published:** 2026-03-24

**Authors:** Teomie S. Rivera‐Miranda, Krystal R. Hans

**Affiliations:** ^1^ Department of Entomology Purdue University West Lafayette Indiana USA

**Keywords:** adult fly emergence, carrion colonization, forensic entomology, insecticides

## Abstract

In forensic entomology, species identification is essential for determining the time of colonization (TOC)—the point at which insects first arrive and begin oviposition on remains. The TOC serves as a proxy for estimating a minimum postmortem interval (minPMI). This study examined factors associated with colonization (e.g., number of individuals, species present and adult emergence rates) of small pig carcasses (*Sus scrofa* L.). A total of 24 carcasses were placed in an open field and treated with low, medium or high concentrations of either bifenthrin‐ or clothianidin‐containing products. In the bifenthrin trial, a negative correlation was observed between insecticide concentration and the number of flies reared, with abundance decreasing as concentration increased; however, this effect was not statistically significant (*p* = 0.11). In contrast, clothianidin exposure significantly influenced blow fly abundance (*p* = 0.05), with no colonization occurring at the highest clothianidin concentration. Analysis of adult fly emergence rates showed no significant effect from bifenthrin (*p* = 0.79) or clothianidin (*p* = 0.63). The species reared differed between trials. During the bifenthrin experiment, four blow fly species were identified—*Phormia regina* (Meigen), *Lucilia illustris* (Meigen), *Lucilia coeruleiviridis* (Macquart) and *Lucilia sericata* (Meigen). In contrast, only *L. sericata* and *L. coeruleiviridis* were recovered from the clothianidin trial. Additionally, dead *Onthophagus hecate* (Panzer) beetles (Scarabaeidae) and *L. coeruleiviridis* adults were observed on or near carcasses exposed to the highest clothianidin concentration. These findings contribute to the understanding of insecticide effects on the blow fly species colonizing remains. Furthermore, it holds forensic significance in cases where insecticide exposure, whether accidental or intentional, is suspected and could influence minPMI estimations during legal investigations.

## Graphical abstract

- High clothianidin concentrations prevented colonization, while bifenthrin showed a non‐significant trend of reduced fly abundance, indicating both insecticides may suppress blow fly activity on treated carcasses.
- Adult emergence rates were unaffected, with no statistically significant differences observed across insecticide treatments, suggesting limited impact on developmental success post‐oviposition.
- Species diversity differed by insecticide, with four blow fly species reared from bifenthrin‐treated carcasses and only two from clothianidin‐treated ones.

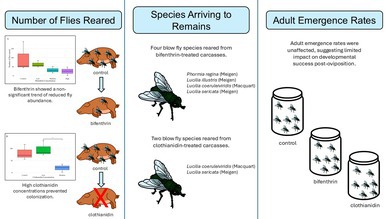

## INTRODUCTION

Forensic entomology is the study of insects and other arthropods in relation to legal investigations (Catts & Goff, 1992). Specifically, the medicolegal branch of forensic entomology focuses on the insects associated with cadavers (human or animal) to estimate the time of insect colonization (TOC) (Catts & Goff, 1992). In cases where external factors such as concealment are absent, TOC can serve as a reliable estimate for the minimum postmortem interval (minPMI), the point at which insects first arrive and begin oviposition on remains (Amendt et al., 2007). Two primary approaches are used to estimate a minPMI: analyzing the developmental stages of insects feeding on the remains, and examining the successional patterns of insect colonization (Maisonhaute & Forbes, 2021; Wells & LaMotte, 2001). Both methods rely on accurate species identification and comparison to known developmental timelines under controlled conditions (Siva Prasad & Aneesh, 2022).

Blow flies (Diptera: Calliphoridae) are typically the first insects to detect and arrive at decomposing remains (Goff, 2010). They are particularly significant in forensic entomology due to their ability to colonize remains within minutes (Goff & Lord, 1994). Female blow flies assess the suitability of remains as a substrate for oviposition and typically lay eggs in the natural body orifices such as the eyes, nose, mouth, and ears (Goff, 2011). The resulting larvae feed on the decomposing tissue, influencing the rate of decomposition (Thakur et al., 2024). Forensic entomological surveys across the United States have documented several blow fly species frequently associated with decomposing remains (Gruner et al., 2007; Pinto et al., 2021; Weidner et al., 2015, 2020). However, species composition varies based on factors such as seasonality, geographic location, and crime scene characteristics (e.g., indoor vs. outdoor settings). For example, a study on the forensically relevant insects associated with human remains in Indiana reported *Phormia regina* (Meigen) as the dominant species in outdoor cases, whereas *Lucilia sericata* (Meigen) was predominant indoors (Weidner et al., 2020). In Connecticut, 96% of blow flies collected from forensic surveys belonged to three species: *P. regina*, *L. sericata* and *Lucilia coeruleiviridis* (Macquart) (Pinto et al., 2021). Another species, *Cochliomyia macellaria* (Fabricius), has been described as a primary colonizer of remains during summer months in Southeastern states, including South Carolina (Tomberlin & Adler, 1998).

In forensic contexts, environmental and biological factors such as temperature, humidity, wind (Campobasso et al., 2001), rainfall (McLeod, 2015), light exposure (e Castro et al., 2011), predation (Harvey, 2024) and competition (Kheirallah et al., 2007) may alter insect colonization patterns and delay TOC. Anthropogenic factors such as burial (Singh et al., 2016), concealment (Lutz et al., 2019) or chemical application can also influence insects and their ability to detect remains.

Chemical‐related fatalities, whether intentional or accidental, are well‐documented. Household chemicals, including gasoline (Bogdanović et al., 2019; Martínez & Ballesteros, 2005) and acids (Schotsmans & de Voor, 2017), have been used in both suicides and homicides. Insecticides are of particular interest due to their potential to repel or kill necrophagous insects. Although insecticide‐related deaths are relatively rare in forensic casework, there is some documentation (De Letter et al., 2002; Klein‐Schwartz & Smith, 1997; Kumar et al., 2009; Menezes et al., 2021). Between 2006 and 2010, 52 insecticide‐related deaths were reported to Poison Control Centers in the United States (Langley & Mort, 2012). Insecticides can be categorized into systemic and non‐systemic types. Systemic insecticides act through ingestion of contaminated plant tissues (Sanchez‐Bayo et al., 2013), while non‐systemic insecticides result in toxicity via direct contact or ingestion of residues shortly after exposure (Du et al., 2025). Bifenthrin, a non‐systemic type I pyrethroid, disrupts the sodium channel gating in both central and peripheral nervous systems, leading to delayed sodium channel closure, paralysis and subsequent death (Dai et al., 2010; Syed et al., 2018). Clothianidin, a systemic neonicotinoid, binds to nicotinic acetylcholine receptors (nAChR) in the insect central nervous system, competing with acetylcholine and disrupting normal neurotransmission, ultimately causing paralysis and death (Uneme, 2011). Previous research has explored the effects of various insecticides on blow fly development, successional patterns and oviposition behavior (Eulalio et al., 2023; Gunatilake & Lee Goff, 1989; Jales et al., 2020, 2021; Yan‐Wei et al., 2010).

The present study aimed to evaluate how bifenthrin and clothianidin insecticide treatments influenced blow fly colonization patterns on pig carcasses during the summer months in Indiana. Specifically, we assessed whether insecticide exposure affected the number of individuals reared, species present and adult emergence rates—key factors in forensic entomology for minPMI estimation. Field experiments involved spraying stillborn pig carcasses with low, medium or high concentrations of each insecticide. Observations of initial oviposition events were recorded, and subsamples of the egg masses were collected and reared to adulthood for species identification. Given that the presence of insecticides can alter blow fly attraction and colonization behaviors, we hypothesized that insecticide exposure would disrupt typical colonization patterns by reducing the abundance of primary colonizers. Specifically, we expected that species typically classified as primary colonizers (e.g., *L. sericata* or *P. regina*) would exhibit delayed or reduced oviposition in treated environments.

## MATERIALS AND METHODS

This study tested two insecticides—bifenthrin and clothianidin—due to their widespread use in residential and agricultural settings (De, 2025; Johnson et al., 2010) and followed the experimental procedures described in Rivera‐Miranda and Hans (2025). The products used in this study contained bifenthrin at a concentration of 7.9% (Talstar® P Professional, EPA Reg. No. 279‐3206, FMC Corporation, Philadelphia, PA) and clothianidin at a concentration of 50% (Arena 50 WDG, EPA Reg. No. 59639‐152, Valent, San Ramon, CA), as active ingredients. Insecticide concentrations were prepared by diluting a stock solution to reflect the maximum recommended application rates for each product. The final concentrations for bifenthrin were 73.51 ppm (low), 735.19 ppm (medium) and 7351.90 ppm (high), while the concentrations for clothianidin were 2.99 ppm (low), 29.95 ppm (medium) and 299.56 ppm (high). Control groups were treated with distilled water. These concentrations were chosen to reflect a range of realistic exposure scenarios, from incidental contact with residual insecticide to deliberate application.

The bifenthrin trial was conducted on July 10, 2023 (mean temperature = 25.3°C ± 2.0°C), while the clothianidin trial was conducted on August 15, 2023 (mean temperature = 25.1°C ± 1.8°C). Both trials were conducted in an open field, bordered by a variety of trees and dense shrubbery (40.428331, −86.949112). Stillborn pig carcasses were sourced from the Purdue University Animal Sciences Research and Education Center (ASREC)‐Swine Unit and stored at −20°C. Two days prior to the experiments, 12 carcasses were thawed in a refrigerator (4°C) and, once thawed, the carcasses were weighed using an electronic scale. The animals used in this study were not euthanized; therefore, ethical approval was not required.

Each carcass was randomly assigned a treatment group and spaced 15 m apart in the field to ensure experimental independence. Each carcass was treated with the corresponding insecticide concentration or water using a controlled application process. Three sprays were applied in a left‐to‐right sweeping motion, delivering 15 mL of solution, in accordance with the product's label instructions. To prevent scavenging and maintain accessibility for insect colonization, all carcasses were enclosed in custom‐made chicken wire cages open at the bottom and secured to the ground using landscape staples. Each insecticide trial involved 12 pig carcasses, with three replicates for each treatment level (e.g., control, low, medium and high). Daily temperature and relative humidity were recorded using a HOBO® External Temperature/RH Sensor Data Logger (MX2301A, Onset Computer Corporation, Bourne, MA).

Following carcass placement, pig remains were monitored at hourly intervals until oviposition occurred. Upon the first observed oviposition event, a subsample of eggs was collected from each carcass using a fine‐tipped paintbrush or soft‐touch forceps to minimize damage. Eggs were collected only once per carcass, and no additional collections were made. This single‐collection approach was chosen intentionally to ensure that all subsamples represented the earliest‐arriving colonizers. After collection, eggs were transferred to mason jars containing fresh, untreated beef liver as a food source and pine shavings as a developmental substrate. The jars were maintained within a fume hood under controlled environmental conditions of 21 ± 0.9°C and 44% ± 5% relative humidity to facilitate development to adulthood. Fresh beef liver was provided ad libitum to support larval growth. After the feeding phase, larvae migrated to the pine shavings at the base of the jars to pupate. Adult emergence rates were calculated as the proportion of pupae that successfully developed into adults, and adult flies were identified to species using a morphological key (Jones et al., 2019) under a stereomicroscope (Stemi 305/508, Zeiss).

### Statistical analysis

To evaluate the impact of insecticide concentration on fly rearing and adult emergence rates, we first assessed the normality of the data using Shapiro–Wilk tests. The results indicated non‐normal distributions across treatment groups, which, combined with the small sample sizes (*n* = 3), justified the use of non‐parametric methods. Consequently, we performed exact permutation tests using 10,000 permutations to generate a null distribution of the Kruskal–Wallis *H*‐statistic, with significance assessed at α = 0.05. Analyses were conducted in R 2024.04.

## RESULTS

From this point on, the term “flies” refers collectively to larvae, pupae and adults, with explicit distinction made when referring specifically to adults. All eggs were reared with the objective of achieving full development to adulthood; however, a subset of specimens did not survive beyond the larval and pupal stages.

**FIGURE 1:**
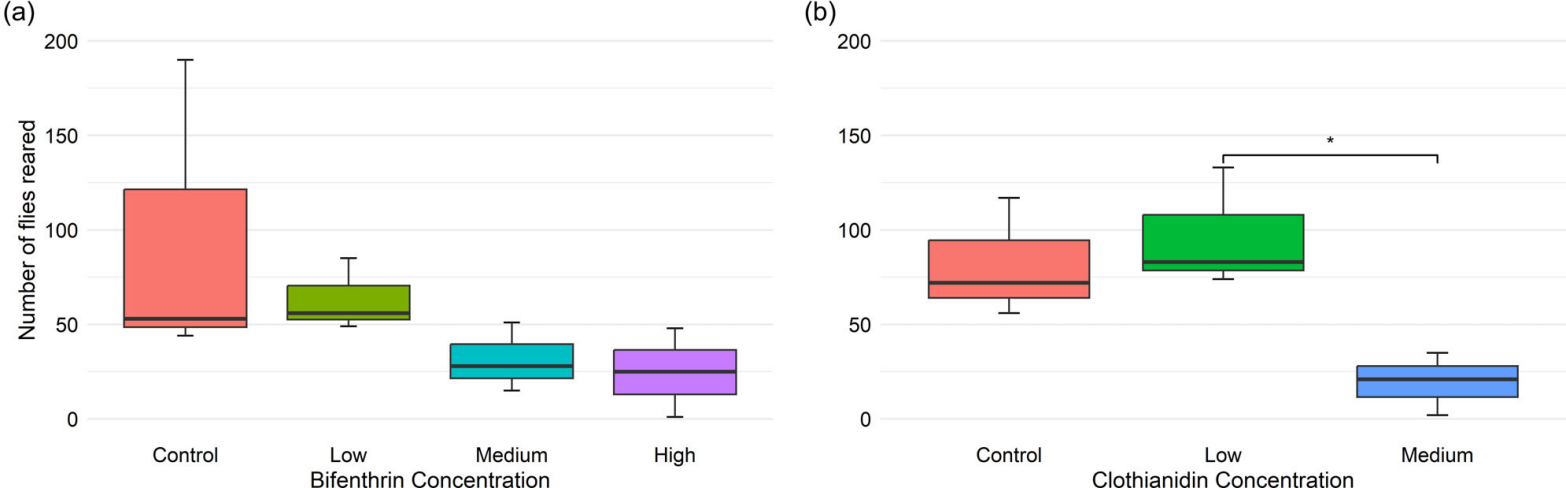
Flies reared from (a) bifenthrin‐ and (b) clothianidin‐treated carcasses. In the bifenthrin trial, fly survival negatively correlated with concentration (Kruskal–Wallis *H* = 6.0256, df = 3, *p* = 0.10). In the clothianidin trial, the low concentration yielded the most flies, followed by the control and medium groups, with a significant effect on total flies reared (Kruskal–Wallis *H* = 5.9556, df = 2, *p* = 0.03).

A total of 645 flies were reared from eggs collected across all pig carcasses treated with bifenthrin. The effect of bifenthrin concentrations resulted in a negative correlation with the control group rearing the highest number of flies, followed by the low, medium and high concentrations (Figure 1a). However, an exact permutation test indicated that these differences were not statistically significant (Kruskal–Wallis *H* = 6.0256, df = 3, *p* = 0.11). The control and high treatments resulted in similar adult emergence rates, followed by the medium and low concentrations (Kruskal–Wallis test, *H* = 1.065, df = 3, *p* = 0.79) (Table 1).

**TABLE 1:** Mean adult fly emergence rates from the bifenthrin‐treated carcasses.

Treatment	Total number of flies	Mean adult fly emergence rates (%)
Control	287	64.1
Low	190	24.2
Medium	94	33.3
High	74	66.7

From the clothianidin‐treated carcasses, 593 flies were reared from the collected eggs. Contrary to the bifenthrin trial, there was no clear trend for clothianidin. The low concentration‐treated carcasses resulted in the highest number of flies reared, followed by the control and the medium treatments (Figure 1b). No insects were obtained from the high concentration group, as no oviposition occurred. Statistical analysis revealed a significant difference among treatments (Kruskal–Wallis test, *H* = 5.9556, df = 2, *p* = 0.05). Pairwise comparisons using Dunn's test indicated that the number of insects reared in the low concentration group was significantly different from that in the medium concentration group (*p* = 0.05), while no other pairwise comparisons yielded statistically significant differences. Although relatively similar, adult emergence rates were highest for the low treatment, followed by the control and medium treatments (Table 2). However, the effects of clothianidin on adult emergence rates were not statistically significant (Kruskal–Wallis test, *H* = 0.9044, df = 2, *p* = 0.63).

**TABLE 2:** Mean adult fly emergence rates from the clothianidin‐treated carcasses.

Treatment	Total number of flies	Mean adult fly emergence rates (%)
Control	245	36.5
Low	290	43.1
Medium	58	33.3

### Number of individuals and species reared

In the bifenthrin trial, a total of 266 adult flies were reared from the initial oviposition events of all experimental groups. Four blow fly species were identified: *Phormia regina* (Meigen), *Lucilia illustris* (Meigen), *Lucilia coeruleiviridis* (Macquart) and *Lucilia sericata* (Meigen). Dead teneral adults—recently emerged from pupae and characterized by soft bodies and lack of pigmentation—accounted for 4.8% of the reared adults, but due to their undeveloped morphological features, these specimens could not be identified to species level. Overall, the relative abundance of species indicated that *P. regina* was the most common species collected across all experimental groups, followed by *L. illustris*, *L. coeruleiviridis* and *L. sericata* (Table 3). In the clothianidin trial, a total of 188 adult flies were reared, and two Calliphoridae species were identified: *L. sericata* and *L. coeruleiviridis*. The control and low treatments had representatives of both species, even though most of the *L. sericata* were reared from the control group. A single *L. coeruleiviridis* fly was reared from the medium treatment. No colonization was observed in the high treatment; therefore, no adults were reared (Table 3).

**TABLE 3:** Relative abundance (%) of species identified from the bifenthrin and clothianidin trials.

Treatment	*Phormia regina*	*Lucilia sericata*	*Lucilia illustris*	*Lucilia coeruleiviridis*
Bifenthrin control	72.9	–	–	–
Bifenthrin low	–	–	1.9	0.8
Bifenthrin medium	–	–	1.9	–
Bifenthrin high	17.3	0.4	–	–
Total bifenthrin^a^	90.2	0.4	3.8	0.8
Clothianidin control	–	81.9	–	0.5
Clothianidin low	–	16.0	–	1.1
Clothianidin medium	–	–	–	0.5
Clothianidin high	–	–	–	–
Total clothianidin	–	97.9	–	2.1

^a^ Teneral adults accounted for 4.8% of adult flies reared.

### Additional observations

Although the insects examined in this study were collected on day 1 of the experiments, observations were extended for an additional 19 days to investigate the effects of insecticides on pig decomposition, which will be discussed elsewhere. During the remaining experimental period, other insect species were observed on the pig carcasses for both insecticide treatments. In the bifenthrin treatment, a distinct blow fly species, *Cochliomyia macellaria* (Fabricius), was observed visiting the carcasses. On day 20, nine additional pupae were collected from pigs treated with the high bifenthrin concentration and later brought to the laboratory for rearing. After 1 week, only two adults emerged, both belonging to the family Sarcophagidae. The remaining pupae were further examined under a stereomicroscope, revealing the presence of small holes, indicative of parasitism by wasps. Upon further analysis, the wasps were identified as belonging to the superfamily Chalcidoidea (Hymenoptera) (Goulet & Huber, 1993). In the clothianidin experiments, *C. macellaria* and Sarcophagidae individuals were also observed visiting the pig carcasses. Additionally, on day 1, *Onthophagus hecate* (Scarabaeidae) beetles and *L. coeruleiviridis* flies were found dead near the pigs treated with the high concentration.

## DISCUSSION

In the bifenthrin trial, a negative correlation was observed between the bifenthrin concentration and the number of flies reared from initial oviposition events, with higher insecticide concentrations resulting in a fewer number of flies. This suggests that bifenthrin may inhibit blow fly colonization, and, in forensic casework, any factor that delays or prevents insect colonization can significantly impact the minimum postmortem interval (minPMI) estimations. Previous studies have reported delays in colonization and a reduction in the number of larvae, pupae and adults on chemically treated remains (Abd El‐bar & Sawaby, 2011; Ghandour & Attia, 2013), reinforcing the potential for insecticides to interfere with entomological evidence.

In the clothianidin trial, the highest number of flies was reared from carcasses treated with the low concentration, while no colonization occurred at the highest concentration. The control group yielded the second highest number of flies, while the medium concentration yielded the lowest number. Similar trends have been observed in other studies, where insecticide‐treated carcasses resulted in a higher number of insects collected (Abd El‐Gawad et al., 2019; Jales et al., 2021). An important consideration when evaluating carcass colonization patterns is female blow fly attraction and oviposition behavior, as females assess potential substrates for their suitability in offspring development (Tomberlin et al., 2011). The presence of insecticides such as bifenthrin and clothianidin may influence carcass acceptance, oviposition behavior and offspring viability.

Adult emergence was also assessed, considering that some egg clutches remained on treated carcasses for 30–40 min prior to collection. In the bifenthrin trial, the high‐concentration group showed unexpectedly high emergence rates, which may reflect sublethal effects that enhance development in surviving individuals, as previously reported in Coleoptera. For instance, sublethal doses (<1 ppm) of neem extract accelerated the development of the red flour beetle (*Tribolium castaneum* Herbst) (Ramachandran et al., 1988), while a low dose of *Tetradenia riparia* extract increased oviposition and adult emergence rates in the Mexican bean weevil (*Zabrotes subfasciatus* Boheman) (Weaver et al., 1992). In the clothianidin trial, although not statistically significant, emergence rates were highest in the low‐concentration group and lowest in the medium group. A potential explanation for the reduced emergence rate in the control treatment may be associated with the rearing protocol. While eggs from each replicate were placed in separate containers, variability in egg clutch size among replicates could have resulted in larval density differences. Higher larval densities, particularly in treatments with a greater number of flies (e.g., the control), could potentially lead to overcrowding and increased competition for resources, ultimately affecting survival and emergence rates (Goodbrod & Goff, 1990; Ireland & Turner, 2006). This particularly underscores the importance of not only considering the environmental factors when analyzing entomological evidence but also considering the experimental factors such as rearing conditions.

Although the two experiments were conducted 1 month apart, differences in the species present in each trial were noted. In the bifenthrin trial, a total of four blow fly species were reared successfully. Of those, *Phormia regina* was the dominant species despite its typical association with cooler climates (Byrd & Castner, 2001). In the clothianidin treatment, only two species were identified: *Lucilia sericata* and *Lucilia coeruleiviridis*, and *L. sericata* was the most abundant. Eggs from both species were collected from the same clutches in the control and low‐concentration groups, supporting previous findings that classify these species as co‐dominant colonizers of pig carcasses during the summer months (Tabor et al., 2005). *P. regina* emerging as the dominant species in the bifenthrin experiment and *L. sericata* prevailing in the clothianidin experiment is consistent with prior studies on the seasonal variation of forensically significant insect species across California, New Jersey and Indiana, where these two species have been documented as predominant species in warm weather conditions (Brundage et al., 2011; Weidner et al., 2015, 2020). The absence or reduced presence of expected primary colonizers observed here suggests that insecticide exposure may selectively inhibit certain species, which further complicates minPMI estimations. In addition to Calliphoridae flies, adults from the family Sarcophagidae were observed near carcasses, but no larval specimens were collected. Parasitic wasps, Chalcidoidea (Hymenoptera), were recovered from Sarcophagidae pupae on the last day of the clothianidin experiment. These wasps are well‐documented parasitoids of Dipteran eggs, larvae and pupae, aligning with their observed presence within the pupal samples (Amendt et al., 2000; Cárcamo et al., 2021; Schuster et al., 2023). Beyond the known repellent and lethal effects of insecticides (Davis, 1985; Deletre et al., 2016; Garson & Winnike, 1968), additional environmental and biological factors may have influenced species diversity and abundance in this study. For instance, rainfall during the clothianidin trial may have altered colonization patterns, delaying oviposition and influencing the species that appeared until environmental conditions improved (Mahat et al., 2009), emphasizing the need for forensic entomologists to consider weather conditions when interpreting insect evidence. Furthermore, interspecific competition can also significantly impact early oviposition events, influencing the subsequent number of species present (Kheirallah et al., 2007; Yang & Shiao, 2012).

Several limitations must be considered when interpreting these findings. First, the study relied on a single egg collection shortly after initial oviposition. This might have influenced the probability of capturing the complete diversity of species present on the remains and restricted the ability to fully assess oviposition delays or repellency effects associated with insecticide exposure. *Cochliomyia macellaria* was observed visiting carcasses treated with both insecticides, but was not represented among the reared specimens. The absence of *C. macellaria* from the reared specimens may indicate a heightened sensitivity to chemical exposure, since this species is recognized as a primary colonizer during the July and August months (Tomberlin & Adler, 1998), and previous studies have reported increased mortality rates and developmental abnormalities following exposure to even low concentrations of insecticides (Chaaban et al., 2017, 2018; Deguenon et al., 2019). Second, the use of small pig carcasses (<2 kg) may have introduced resource limitations, influencing interspecific competition and favoring some species over others (Lutz et al., 2022). This could have skewed species representation and abundance. Third, the inability of some species to develop under laboratory conditions likely contributed to their underrepresentation. Specifically, *Lucilia coeruleiviridis* has been reported to experience low survival rates in controlled environments (Bagsby & Hans, 2024; Weidner et al., 2014). While a limited number of *L. coeruleiviridis* specimens were successfully reared in both trials, it is likely that this species was present in most, if not all, treatments but failed to develop due to suboptimal laboratory conditions. This is further supported by the high number of dead larvae recovered across all treatments.

Although insecticides are not frequently encountered in homicide cases, their forensic relevance lies in their potential to alter decomposition and insect colonization patterns. Whether applied intentionally to conceal remains or present due to environmental contamination, surface‐applied insecticides like bifenthrin and clothianidin can disrupt the entomological timeline used to estimate the minPMI (Rivera‐Miranda & Hans, 2025). Previous case reports have documented the use of chemicals, including insecticides, which have resulted in delayed decomposition (Kumar et al., 2009) and possibly on insect detection and colonization of remains. Moreover, the widespread availability of bifenthrin and clothianidin in household and agricultural products increases the likelihood of their presence in forensic contexts.

This study provides insights into the effects of bifenthrin and clothianidin on the colonization success and adult emergence of forensically relevant blow fly species associated with carrion decomposition. The results of this study support our hypothesis that insecticide exposure disrupts typical colonization patterns by reducing the number of primary colonizers. Moreover, the detection of dead *L. coeruleiviridis* and *Onthophagus hecate* (Panzer, 1794) beetles (Coleoptera: Scarabaeidae) near clothianidin‐treated carcasses also suggests that high insecticide concentrations may exert lethal effects beyond Dipteran taxa. Overall, the findings emphasize the need for forensic entomologists to consider chemical interference when interpreting insect evidence, especially in cases where colonization appears delayed, atypical or absent.

## AUTHOR CONTRIBUTIONS

**Teomie S. Rivera‐Miranda:** Conceptualization; investigation; writing – original draft; methodology; writing – review and editing. **Krystal R. Hans:** Conceptualization; writing – review and editing; supervision; formal analysis.

## FUNDING INFORMATION

American Academy of Forensic Sciences and Purdue University.

## CONFLICT OF INTEREST STATEMENT

The authors declare no conflicts of interest.

## Data Availability

The data that support the findings of this study are openly available in *Insect numbers and adult emergence* at https://doi.org/10.6084/m9.figshare.30508421.v2, (Rivera‐Miranda, 2025).
